# Post-Transcriptional Regulation of Cadherin-11 Expression by GSK-3 and β-Catenin in Prostate and Breast Cancer Cells

**DOI:** 10.1371/journal.pone.0004797

**Published:** 2009-03-10

**Authors:** Anne K. Farina, Yong-Sik Bong, Carolyn M. Feltes, Stephen W. Byers

**Affiliations:** Lombardi Comprehensive Cancer Center and Departments of Oncology and Biochemistry, Molecular and Cellular Biology, Georgetown University School of Medicine, Washington, D. C., United States of America; University of Helsinki, Finland

## Abstract

**Background:**

The cell-cell adhesion molecule cadherin-11 is important in embryogenesis and bone morphogenesis, invasion of cancer cells, lymphangiogenesis, homing of cancer cells to bone, and rheumatoid arthritis. However, very little is known about the regulation of cadherin-11 expression.

**Methodology/Principal Findings:**

Here we show that cell density and GSK-3β regulate cadherin-11 levels in cancer cells. Inactivation of GSK3β with lithium chloride or the GSK3 inhibitor BIO and GSK3β knockdown with siRNA repressed cadherin-11 mRNA and protein levels. RNA Polymerase II chromatin immunoprecipitation experiments showed that inhibition of GSK3 does not affect cadherin-11 gene transcription. Although the cadherin-11 3′UTR contains putative microRNA target sites and is regulated by Dicer, its stability is not regulated by GSK3 inhibition or density. Our data show that GSK3β regulates cadherin-11 expression in two ways: first a β-catenin-independent regulation of cadherin-11 steady state mRNA levels, and second a β-catenin-dependent effect on cadherin-11 3′UTR stability and protein translation.

**Conclusions:**

Cadherin-11 mRNA and protein levels are regulated by the activity of GSK3β and a significant degree of this regulation is exerted by the GSK3 target, β-catenin, at the level of the cadherin-11 3′UTR

## Introduction

Cadherin-11, also known as OB-cadherin was first identified in mouse osteoblasts and is normally expressed in cells with a mesenchymal phenotype and of mesodermal origin, including the mesenchyme of the kidney and brain during development [1]. Cadherin-11 is also expressed in cartilage synoviocytes and is an important mediator of the synoviocyte reaction that characterizes rheumatoid arthritis [2], [3]. The cadherin-11 knockout mouse exhibits bone and behavioral abnormalities and is resistant to induction of rheumatoid arthritis [2], [3]. Additionally, cadherin-11 is expressed in several types of cancer including breast and prostate cancers, osteosarcoma, and colon cancer [4]–[8]. In these cancers cadherin-11 expression is associated with the most aggressive and most metastatic cancer cells.

Little is known about the regulation of cadherin-11 expression. TGFβ1 regulates cadherin-11 in cultured extravillous cytotrophoblasts, and progesterone, but not 17-beta-estradiol, regulates cadherin-11 in cultured endometrial stromal cells undergoing decidualization [9], [10]. During development cadherin-11 is expressed in cranial neural crest cells of the developing *Xenopus* embryo, and XWnt-8 overexpression increases Xcadherin-11 mRNA in *Xenopus* animal cap cells [11], [12].

The principle pathway associated with canonical Wnt signaling involves inhibition of GSK3. GSK3 is intricately involved in many pathways essential to cellular function. Other mechanisms that also negatively regulate GSK3β include GSK3β phosphorylation on Ser9 by Akt and inhibition by LiCl treatment and 6-bromoindirubin-3′-oxime (BIO) treatment [18]–[20]. In the absence of canonical Wnts, GSK3 binds and phosphorylates β-catenin, in a multi-protein complex including axin, adenomatous polyposis coli (APC), and protein phosphatase 2A. Phosphorylation by GSK3β results in β-catenin protein degradation [13]–[15]. In the presence of canonical Wnts, activated Disheveled prevents GSK3-dependent phosphorylation of β-catenin [17].

In this study we use breast cancer cells with low levels of endogenous activated GSK-3 and β-catenin and prostate cancer cells with constitutively activated GSK-3 and β-catenin to show that, GSK3β regulates cadherin-11 expression in two ways. The first mechanism is through β-catenin-independent regulation of steady state cadherin-11 mRNA levels. The second mechanism involves a β-catenin-dependent effect on cadherin-11 3′UTR stability and protein translation.

## Results

### Cell density and inhibition of GSK3β regulate cadherin-11 mRNA and protein expression

**Figure 1:**
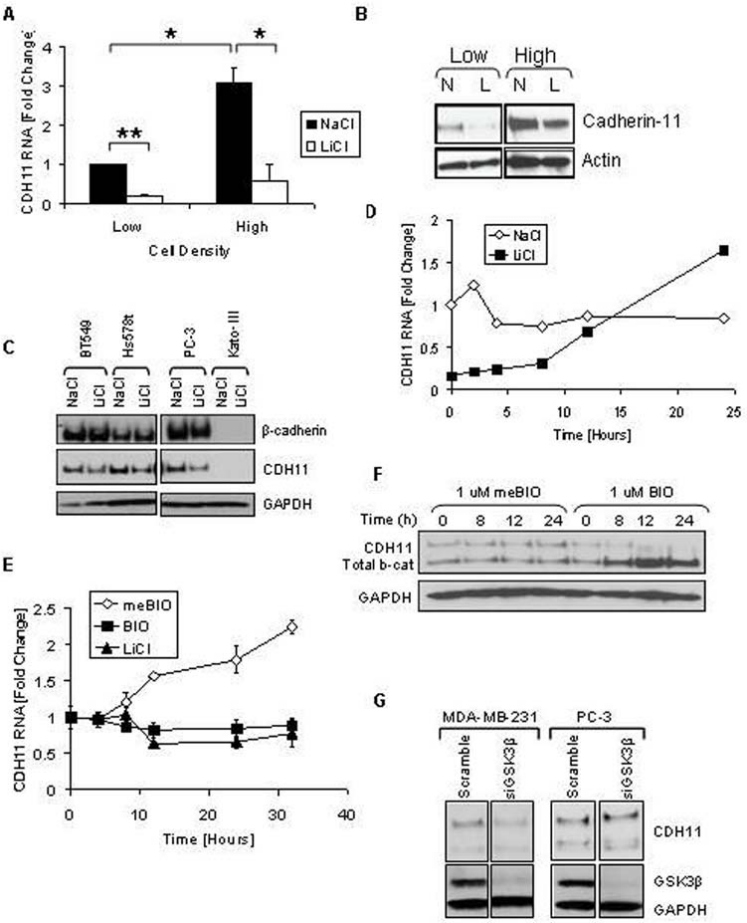
Density increases cadherin-11 and GSK3β inhibitors decrease cadherin-11 expression. A and B: MDA-MB-231 cells were plated at two densities, 50% and 90% confluency, called low and high, respectively. The cells were allowed to grow overnight in serum-free medium. 24 hours after plating cells were treated with 20 mM NaCl (N) or 20 mM LiCl (L). 24 hours after treatment, RNA and protein were collected for real-time PCR (A) and Western (B) analysis. C: Cancer cells BT549, Hs578T, PC-3, and Kato-III were plated at a medium density and allowed to adhere. Cells were then treated with 20 mM LiCl or NaCl control. 48 hours after transfection protein was collected for Western blot analysis. D: MDA-MB-231 cells were plated at a medium density, serum starved overnight, and treated with 20 mM NaCl or 20 mM LiCl. 24 hours after treatment, the cells were washed once with PBS and maintained in serum-free medium containing only 20 mM NaCl. RNA was collected at the times indicated for real-time PCR analysis. E and F: MDA-MB-231 cells were plated at a medium density and serum starved overnight. 16 hours after plating, the cells were treated with 1 μM meBIO (control), 1 μM BIO, or 20 mM LiCl. RNA (E) or protein (F) was collected at the designated times and analyzed using real-time PCR (E) and Western blot analysis (F). G: MDA-MB-231 and PC-3 cells were transfected with either non-specific scrambled siRNA (siScramble), or siRNA directed against GSK3β (si GSK3). 48 hours after transfection protein was isolated for Western blot analysis.

MDA-MB-231 cells, a mesenchymal-like breast cancer cell line that expresses cadherin-11, have undetectable levels of other cadherins and low levels of activated β-catenin. In pilot experiments we noticed that cadherin-11 expression varied noticeably depending on the number of cells plated. Figure 1 shows that cells grown at higher cell densities had markedly increased cadherin-11 mRNA (Figure 1A) and protein (Figure 1B) expression on a per cell basis compared to those grown at low density. In *Xenopus laevis*, cadherin-11 is regulated by xWnt8, although it is not known if the cadherin-11 gene is a direct transcriptional target or if either the canonical or non-canonical pathways are involved [11]. To test if the canonical Wnt pathway regulates cadherin-11 in MDA-MB-231 cells, we treated cells with lithium chloride (LiCl), an inhibitor of GSK3β. LiCl treatment repressed cadherin-11 mRNA and protein levels and blocked the effects of increased cell density (Figure 1A, B). Cadherin-11 protein levels were also inhibited by LiCl in three other cell-lines (BT549, Hs578t and PC-3 Figure 1C). Remarkably, N-cadherin, which is endogenously expressed by these cell-lines and not by MDA-MB-231 cells, was unaffected by LiCl, strongly indicating that inhibition of GSK3β specifically regulated cadherin-11 expression. If the effects of LiCl are due to transient inhibition of GSK3β we anticipated that removal of LiCl should reverse its effects on cadherin 11. Figure 1D shows that this is indeed the case as cadherin-11 levels returned to control values 12 hours following LiCl washout. To confirm that the effects of LiCl were related to inhibition of GSK3β, we treated cells with 6-bromoindirubin-3′-oxime (BIO), a small molecule kinase inhibitor selective for GSK3α/β (Figure 1E, F). Inhibition of GSK3β with BIO stabilized β-catenin, and like LiCl, reduced cadherin-11 mRNA and protein accumulation. Furthermore, siRNA directed against GSK3β mRNA decreased cadherin-11 protein expression in the absence of either LiCl or BIO in MDA-MB-231 but not in PC-3 cells (Figure 1G). Therefore, GSK3β knockdown had little effect on cadherin-11 levels in PC-3 cells (Figure 1G). Unlike MDA-231 cells, PC-3 cells have high levels of nuclear activated β-catenin as a result of a PTEN mutation and constitutive inactivation of GSK3β [21]. Consequently, removal of GSK3β in these cells may have less effect as it is already inactivated.

### Recruitment of RNA polymerase II to the cadherin-11 gene is unaffected by

**Figure 2:**
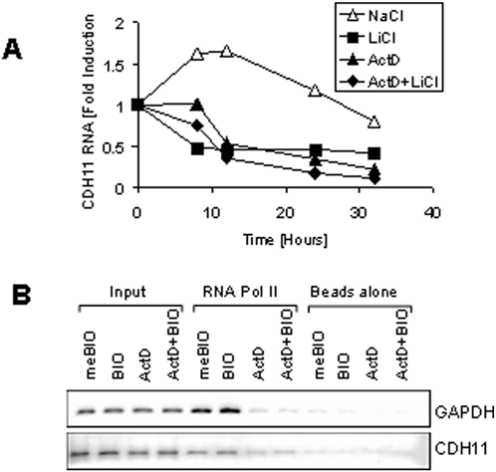
Transcriptional mechanism of cadherin-11 regulation. A: MDA-MB-231 cells were grown at a medium density for 16 hours in the absence of serum. The cells were then pretreated with 5 μg/ml actinomycin D or an equivalent volume of ethanol (untreated). 30 minutes later the cells were treated with either 20 mM NaCl (control) or LiCl. At the indicated time points, RNA and protein were collect for real-time PCR analysis. B: MDA-MB-231 cells were treated with 20 mM NaCl or LiCl for 24 hours. Genomic DNA was then harvested for RNA polymerase II ChIP analysis followed by PCR specific to GAPDH and cadherin-11.

To test if GSK3β regulates cadherin-11 expression at the level of transcription, MDA-MB-231 cells were treated with the RNA polymerase II inhibitor, actinomycin D and were compared to the results of cells treated with LiCl. Figure 2A shows that cadherin-11 mRNA levels declined following either LiCl or actinomycin D treatment. However, in this experiment, and on other occasions, we noticed that LiCl treatment repressed cadherin-11 mRNA levels more rapidly than actinomycin D suggesting an additional non-transcriptional level of regulation. If the effects of GSK3β inhibition on cadherin-11 mRNA have a transcriptional component, we would anticipate that recruitment of RNA polymerase II to the cadherin-11 gene to decrease. To test this hypothesis, we carried out RNA polymerase II chromatin immunoprecipitation assays (Pol II ChIP) using cadherin-11 specific primers which amplify a portion of intron 1 (see Materials and Methods). Figure 2B shows that, as expected, actinomycin D markedly reduced the amount of Pol II recruited to both GAPDH and the cadherin-11 gene. However, even though cadherin-11 mRNA levels are markedly reduced by treatment of cells with BIO (Figure 1) it did not affect Pol II recruitment to the cadherin-11 gene (Figure 2B). In other experiments we found that members of the Snail/Slug family, transcriptional repressors known to be regulated by GSK3β and by β-catenin are not involved in cadherin-11 repression (Figure S1) [22]. Taken together these experiments indicate that inhibition of GSK3β by LiCl or BIO influences cadherin-11 mRNA and protein levels by mechanisms largely independent of transcriptional repression.

### The cadherin-11 3′untranslated region (UTR) is highly conserved and destabilizing in cadherin-11 expressing cells

**Figure 3:**
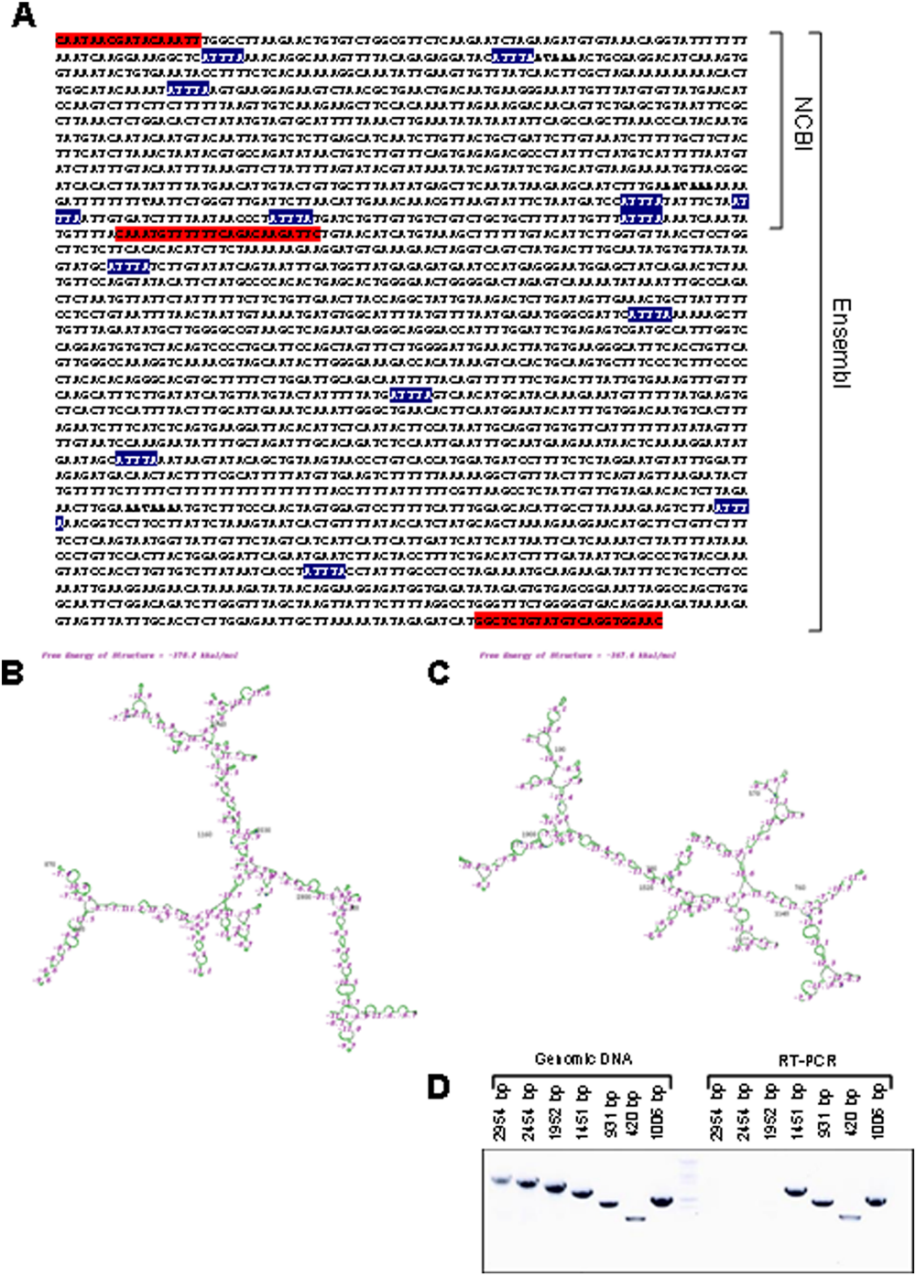
*In silico* evaluation of the cadherin-11 3′UTR. A: Sequence of cadherin-11 3′-UTR according to the Ensembl database (NM_001797). Bolded sequences indicate the poly-A signals and site respectively. Blue highlighted sequences indicate Shaw-Kamens, destabilizing sequences. The first two red highlighted sequences indicate the primers used to design pGL3-CDH11-3′UTR NCBI, as denoted by the bracket. The first and last red highlighted sequences indicate the primers used to design pGL3-CDH11-3′UTR Ensembl, as denoted by the bracket. B: Predicted secondary structure of the Ensembl cadherin-11 3′UTR (as predicted by GeneBee). C: Predicted secondary structure of the Ensembl E-cadher 3′UTR (as predicted by GeneBee). D: RT-PCR of PC3 RNA using primers designed approximately every 500 bp.

In many situations regulation of mRNA stability and its capacity for translation are mediated by the 3′-untranslated region (UTR). If the cadherin-11 3′-UTR is important in the normal regulation of cadherin-11 expression we might expect it to be conserved across species. Table 1 shows the percent identity of the human cadherin-11 and E-cadherin transcripts and 3′-UTRs compared with other species. Remarkably for a region that is not translated the cadherin-11 3′-UTR was almost completely conserved across species (94% human with mouse). In contrast the E-cadherin-3′UTR is poorly conserved (17% human with mouse). Analysis of the secondary structure of this region of both the cadherin-11 and E-cadherin 3′UTRs revealed a high degree of stem loop structures (Figure 3B and Figure 3C). These observations strongly suggest that the cadherin-11 3′UTR has a conserved regulatory function.

**Table 1:** Untranslated region and coding sequence alignment.

ClustalW Nucleotide Sequence Alignment compared to *H. sapiens*						
Species	E-cadherin protein coding sequence	E-cadherin 3′UTR	Cadherin-11 protein coding sequence	Cadherin-11 5′UTR	Cadherin-11 3′UTR (NCBI)	Cadherin-11 3′UTR (Ensembl)
*M. musculus*	79	17	89	84	94	85
*R. norvegicus*	80	27	90	76	94	84
*P. troglodytes*	99	98	99	98	99	99
*G. gallus*	64	N/A	80	59	86	68

The NCBI database identifies a region of the cadherin 3′UTR which spans approximately 1 kb and includes a traditional poly-A signal and site. However, the Ensembl database identifies a cadherin-11 3′UTR of roughly 3 kb which includes a second poly A signal 2363 bp from the stop codon (Figure 3A). The poly-A signal sequences (AAUAAA) within the 3′UTR are bolded in Figure 3A. Blue highlighted sequences indicate Shaw-Kamens sequences, also known as AU-rich elements (AREs [Shaw and Kamen, 1986]). Many microRNA binding elements also exist in the cadherin-11 3′UTR (too numerous to indicate) as determined by ARGONAUTE 2 and miRanda. To determine the actual length of the cadherin-11 3′UTR, total RNA was isolated from PC3 metastatic prostate cancer cells (Figure 3D), MDA-MB-231 cells (data not shown), and MRC5 lung fibroblast cells (data not shown) for analysis. Primers were designed approximately every 500 bp of the Ensembl sequence to make progressively shorter PCR products. In all three cell lines primers designed to produce a PCR product of 1451 bp amplify a product, but the primers designed to make a product of 1952 bp do not (Figure 3D). Although, this 501bp does not include a poly-A signal or addition site one does exist at position 2363 indicating that this longer transcript may be an alternate 3′-UTR.

To investigate the function of the cadherin-11 3′-UTR, it was cloned from MDA-MB-231 genomic DNA. A series of stop codons were included at the 5′-end of the construct followed by the DNA sequence directly 3′ to exon 13, the 3′-UTR. Both the NCBI and Ensembl 3′UTRs were cloned using the red highlighted primer sequences in Figure 3A into a luciferase reporter vector and named luc-CDH11 3′UTR NCBI and luc-CDH11 3′UTR Ensembl, respectively.

**Figure 4:**
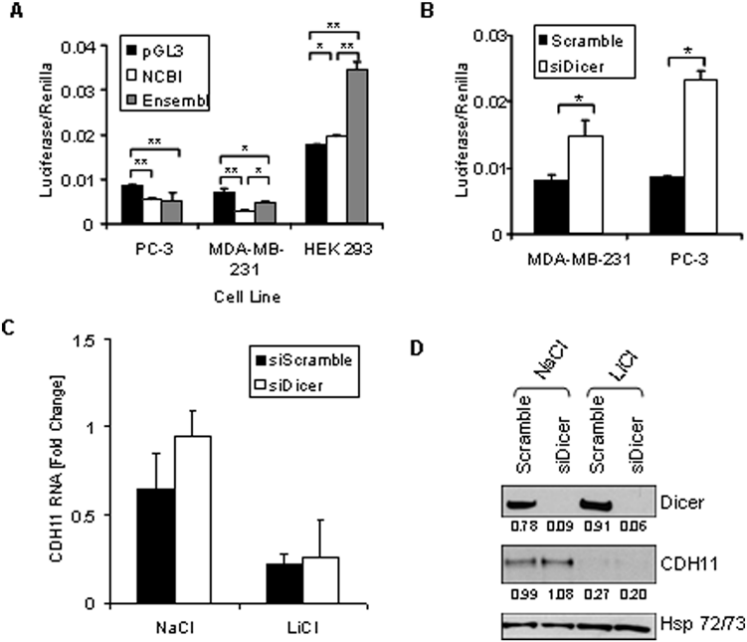
The stabilizing and destabilizing effects of the cadherin-11 3′UTR. A: PC-3, MDA-MB-231, and HEK 293 cells were transfected with pGL3-Promoter, pGL3-CDH11-3′UTR NCBI, or pGL3-CDH11-3′UTR Ensembl and pCMV-Renilla. 48 hours after transfection, cells were lysed using passive lysis buffer and luciferase activity was analyzed. (* indicates a p-value >0.01; ** indicates a p-value >0.001) B: MDA-MB-231 and PC-3 cells were transfected with pGL3-CDH11 3′-UTR and pCMV-Renilla along with either non-specfic siRNA (siScramble) or siRNA directed against Dicer (siDicer). 48 hours after transfection, cells were lysed using passive lysis buffer and luciferase activity was analyzed. (* indicates a p-value >0.05) C and D: MDA-MB-231 cells were transfected with non-specific siRNA (siScramble), or siRNA directed against Dicer (si Dicer). Cells were collected for real-time PCR (C) and Western blot (D) analysis 72 hours after transfection.

In cadherin-11 positive PC-3 and MDA-231 cells both luc-CDH11 3′UTR NCBI and luc-CDH11 3′UTR Ensembl were significantly less active than pGL3, which has the SV40 poly A addition signal and site (Figure 4A). In HEK293 cells which do not express cadherin-11, luc-CDH11 3′UTR Ensembl was more active than either control vector or luc-CDH11 3′UTR NCBI (Figure 4A). These data indicate that the both cadherin-11 3′UTRs confer destabilization properties when expressed in cells which normally make cadherin-11. In contrast, in HEK293 cells, which do not normally make cadherin-11, luc-CDH11 3′UTR Ensembl but not luc-CDH11 3′UTR NCBI is stabilizing.

### The activity of the cadherin-11 (UTR) is regulated by Dicer

To investigate if microRNA influenced the activity of luc-CDH11 3′-UTR we knocked down Dicer, which is essential for the production of microRNAs. Removal of Dicer significantly increased the activity of luc-CDH11 3′-UTR indicating that microRNAs regulate the endogenous activity of the cadherin-11 3′-UTR (Figure 4B). Consistent with a role for miRNA, several putative miRNA binding sites exist in this region, however, none of these predicted miRNAs was regulated by inhibition of GSK3β (Figure S2). These data demonstrate that although miRNAs can regulate luc-CDH11 3′-UTR this is not the mechanism whereby GSK3β inhibition regulated cadherin-11 mRNA levels. Unlike siRNAs, which always direct mRNA degradation, miRNAs often regulate protein translation without affecting mRNA levels [23]–[25]. To test this we examined the effects of Dicer knockdown on cadherin-11 protein and mRNA levels. Figures 4C and 4D show that even in the face of Dicer knockdown, endogenous cadherin-11 mRNA and protein levels were unchanged and the repressive effects of LiCl were not reversed. These data indicate that although the basal activity of the cadherin-11 3′UTR is affected by Dicer knockdown, cadherin-11 mRNA and protein levels are not.

### β-catenin regulates the activity of the cadherin-11 3′UTR, steady state mRNA levels and cadherin-11 protein translation in PC-3 cells

**Figure 5:**
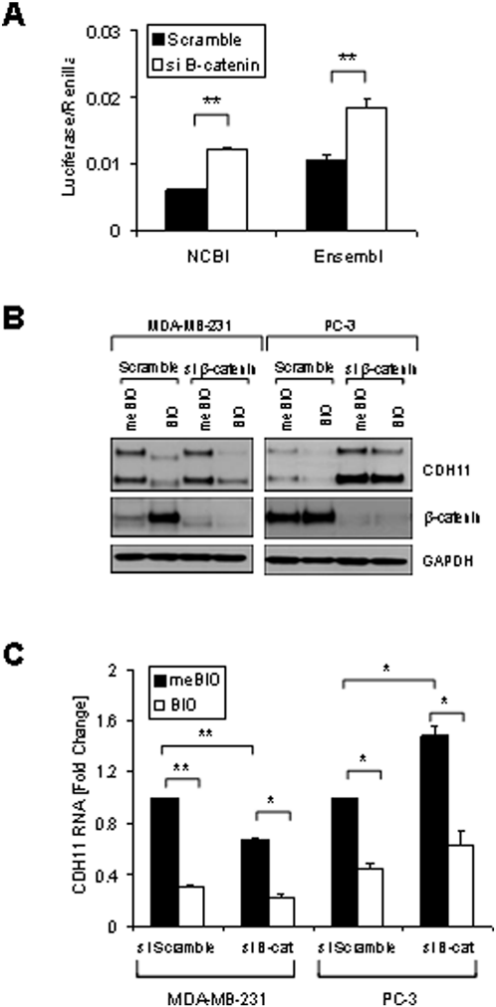
β-catenin as a regulator of cadherin-11 expression. A: PC-3 cells were transfected with pGL3-CDH11-3′UTR NCBI or pGL3-CDH11-3′UTR Ensembl and pCMV-Renilla and along with either non-specific siRNA or siRNA directed against CTNNB1. 48 hours after transfection cells were were lysed using passive lysis buffer and luciferase activity was analyzed. Luciferase activity was normalized to renilla activity. (** indicates a p-value >0.001). B: MDA-MB-231 and PC-3 cells were transfected with either non-specific scrambled siRNA (siScramble) or siRNA directed against CTNNB1 (si β-cat). 24 hours after transfection cells were treated with 1 μM BIO or meBIO (control). 48 hours after transfection protein was isolated for Western blot analysis. C: MDA-MB-231 and PC-3 cells were transfected with either non-specific scrambled siRNA (siScramble) or siRNA directed against CTNNB1 (si β-cat). 24 hours after transfection cells were treated with 1 μM BIO or meBIO (control). 48 hours after transfection RNA was isolated for real-time PCR analysis. (* indicates a p-value >0.05; ** indicates a p-value >0.005)

Perhaps the best known substrate of GSK3β is β-catenin and many studies have shown that Wnt-mediated inhibition of GSK3β stabilizes and activates cytoplasmic β-catenin [17], [26]. Although the effects of inhibition of GSK3β on cadherin-11 are not mediated transcriptionally it is possible that stabilization of β-catenin may repress cadherin-11 mRNA and/or protein levels using an alternative mechanism. To test this PC-3 cells, which normally express high levels of activated β-catenin, were transfected with luc-CDH11 3′UTR Ensembl or luc-CDH11 3′UTR NCBI and treated with β-catenin siRNA [21]. Like Dicer knockdown, knockdown of β-catenin significantly increased the activity/stability of both cadherin-11 3′UTR reporters (Figure 5A). However, in contrast to Dicer knockdown, removal of β-catenin significantly increased cadherin-11 protein and steady state mRNA levels in PC-3 cells (Figure 5C).

## Discussion

The majority of patients dying from breast or prostate cancer have metastases to the skeleton. Although bone metastases are incurable, patients survive several years suffering serious morbidity, including fractures, spinal cord compression, severe bone pain, and hypercalcemia [5], [27]. In the adult, cadherin-11 is strongly expressed in bone as well as certain metastatic cancers, particularly those inclined to metastasize to bone [4], [6], [27]. Once in the bone, it is possible that these cadherin-11-expressing tumor cells activate either osteoclasts or osteoblasts, depending on the type of cancer metastasis, leading to bone remodeling. As the bone is remodeled, growth factors are released into the matrix further stimulating the tumor cells by creating a fertile environment for growth. These changes are reminiscent of those that occur during rheumatoid arthritis, a disease that is also characterized by an important role for cadherin-11 in the activated synoviocyte [2], [3]. Given its role in these important diseases we sought to determine how cadherin-11 expression is regulated. In the present study we show that cadherin-11 mRNA and protein levels are increased by high cell density and are markedly regulated by the activity of the ubiquitous serine/threonine kinase GSK3β. Remarkably, a significant degree of regulation is exerted by the GSK3β target, β-catenin, at the level of the cadherin-11 3′UTR.

### A role for GSK3β and β-catenin in cadherin-11 mRNA and protein homeostasis

The best studied mechanism by which GSK3β is regulated is Akt-dependent serine phosphorylation. Growth factors, such as insulin, activate the phosphatidylinositol 3-kinase (PI3K)/Akt pathway. PI3K converts PIP_2_ to PIP_3,_ a critical step necessary for the activation of PKB/Akt. PTEN is a phosphatase commonly mutated in cancers that is necessary for the dephosphorylation of PIP_3_ thereby inhibiting Akt activation. Once activated Akt acts on many cellular proteins including mTOR, caspase 9, Bad, IKK, and GSK3β. Akt phosphorylates GSK3β on serine 9 and GSK3α on serine 21 to inhibit their kinase activity. Understanding GSK3β regulation by the Akt pathway is important to the present study because a PTEN mutation in PC-3 prostate cancer cells results in activated Akt, GSK3β inhibition, and activation of endogenous β-catenin [28]. Importantly, GSK3β is central to the promotion of inflammation in various inflammatory diseases including colitis and arthritis [29], [30]. The second well-defined mechanism of GSK3β regulation is the canonical Wnt pathway in which inhibition of GSK3β and stabilization of β-catenin results in marked changes in gene expression. Although many studies have observed that Wnt-GSK3β-mediated activation of β-catenin results in accumulation of nuclear and cytoplasmic β-catenin, most studies concentrate on the function of β-catenin as a transcriptional co-activator of TCF, and more recently nuclear receptors, in the nucleus [31]–[33]. However, β-catenin has significant homology to the RNA binding protein pumilio and several recent studies have pointed to an important role for β-catenin in post-transcriptional regulation [34]. For example, β-catenin regulates VEGF-D, cyclooxygenase-2 and cyclin D1 mRNA stability potentially by interaction with their respective 3′-UTRs [35]-[38]. β-catenin can also regulate alternative splicing of estrogen receptor β (ERβ) in colon cancer cells [35]–[38]. The ribosome-associated factor eIF6 associates with β-catenin and the complex may have a role in translational regulation in concert with miRNAs [39], [40]. However, although the endogenous activity of the cadherin-11 3′-UTR is regulated by Dicer, cadherin-11 mRNA and protein regulation by GSK3β are not, ruling out a role for miRNAs in this process. Our data show that the activity of the cadherin-11 3′-UTR is regulated by β-catenin in cells which normally express activated β-catenin such as PC-3 prostate cancer cells. Taken together these data point to an important role for GSK3β and β-catenin in cadherin-11 mRNA and protein homeostasis and may lead to the development of therapeutics for diseases such as, metastatic prostate and breast cancer and rheumatoid arthritis, that are characterized by elevated cadherin-11. Indeed inhibition of GSK3β or ablation of the GSK3β gene ameliorates inflammation dependent arthritis [29].

## Materials and Methods

### Materials and Reagents

LiCl (L9650), NaCl (S3014), and actinomycin D (A1410) were obtained from Sigma-Aldrich (Germany), meBIO (361556) and BIO (361550) from Calbiochem (San Diego, CA), anti-cadherin-11 (5B2H5) from Invitrogen (32-1700, Carlsbad, CA), anti-β-catenin antibody from BD Biosciences (610154, San Jose, CA), Dicer antibody from Abcam (ab14601, Cambridge, MA), anti-GSK3β antibody from Cell Signaling Technologies (9315, Boston, MA), anti-FLAG (M2) was from Sigma (F3165, Germany), anti-GAPDH was from Research Diagnostics Inc (TRK5G4-6C5, Flanders, NJ), anti-Snail (H-130) (sc-28199) antibody from Santa Cruz. The anti-eIF6 antibody S13 was a generous gift from Dr. Biffo and generated against a C-terminus peptide of eIF6 [40], [41]. Small interfering RNA (siRNA) reagent (SMART pool) for human CTNNB1 (M-003482-00), Dicer (M-003483-00), and GSK3β (M-003010-03), were purchased from Dharmacon (Lafayette, CO). Non-specific (Scramble) siRNA was generated using forward: (5′-AAGCTCCTATAGCGTATGGTGCCTGTCTC-3′) and reverse:

(5′-CACCATACGCTATAGGAGCTTCCTGTCTC-3′) primers and the Silencer siRNA Construction kit (AM1620, Ambion, Austin, TX).

### Expression Vectors

Plasmid DNA encoding wild type (WT) β-catenin was used as previously described and DNA encoding wild type Snail was a kind gift from Dr. Mien-Chie Hung [15], [22]. Dominant negative (DN) Snail was generated by PCR from the wild type Snail construct using primers described in Yamasaki *et al.* forward: (5′-CGGGATCCACTATGGCCTTCAACTGCAAATACTG-3′) and reverse: (5′-CGCTCGAGGCGGGGACATCCTGAGCA-3′) and cloned into pCMV-Tag2 (Stratagene, La Jolla, CA) using *BamHI* and *XhoI* restriction sites [42]. Cadherin-11 3′-UTR fragment was cloned from genomic DNA obtained from MDA-MB-231 cells using Expand High Fidelity PCR System (11732641001, Roche, Indianapolis, IN) according to the manufacturer's protocol. For amplification of the CDH11 3′UTR NCBI forward:

(5′-TGCTAGCTAAGTAAGTAACAATAACGATACAAATTT-3′) and reverse:
(5′-CCGGATCCACGCGTGAATCTTGTCTGAAAAAACATTTG-3′) primers were used. For amplification of the CDH11 3′UTR Ensembl forward:
(5′-TGCTAGCTAAGTAAGTAACAATAACGATACAAATTT-3′) and reverse:
(5′-GGATCCACGCGTGTTCCACCTGACATACAGAGCC-3′) primers were used. These PCR products were ligated in place of the SV40 3′UTR of pGL3-Promoter (Promega, Madison, WI).

### Cell Culture and Transfection

MDA-MB-231, Hs578T, BT549 breast cancer cells, PC-3 prostate cancer cells, and HEK293 cells were maintained in Dulbecco's Modified Eagle's Medium (DMEM) supplemented with 5% fetal bovine serum (FBS) in 5% CO_2_ incubator at 37°C. All transient transfections of plasmid DNA and siRNA in MDA-MB-231 and PC-3 cells were performed with Amaxa electroporation system (Amaxa, Inc, Geithersburg, MD) according to the manufacturer's protocol. Transient transfections for immunofluorescence were preformed using Lipofectamine 2000 Transfection Reagent (11668-019, Invitrogen, Carlsbad, CA) and luciferase analysis using Lipofectamine 2000 (Invitrogen) for PC-3 cells, ProFection Mammalian Transfection System—Calcium Phosphate (E1200, Promega, Madison, WI) for MDA-MB-231 cells, and FuGENE 6 Transfection Reagent (11814443001, Roche, Switzerland) for HEK293 cells.

### RNA isolation

#### RNA isolation (mRNA)

MDA-MB-231 or PC-3 cells were incubated in the presence or absence of LiCl (20mM) or BIO (1 μM) for 24 h. RNA was isolated using Trizol (15596-018, Invitrogen) combined with RNAeasy (74106, Qiagen, Valencia, CA) according to the manufacturer's instructions.

#### Total RNA isolation (microRNA)

MDA-MB-231 cells incubated in the presence or absence of 20 mM LiCl for 24 hours. Total RNA was isolated using the miRVana RNA isolation kit (AM1562, Ambion).

### Real time quantitative PCR

#### mRNA

Relative quantitation was used to evaluate the raw data obtained from real-time PCR (7900 HT real time PCR system, Applied Biosystems, Foster City, CA). Single-stranded cDNA was prepared using TaqMan Reverse Transcription Reagents (N808-0234, Applied Biosystems) following the manufacturer's protocol. TaqMan Universal PCR Master Mix (4304437, Applied Biosystems) was used for all reactions. All primer/probe mixes (CDH11 Hs00156438_m1, GAPDH Hs99999905_m1) were obtained from Applied Biosystems and performed in triplicate. The samples were analyzed using the delta-delta Ct method of analysis [43]. The final value obtained was a measure of the fold change in gene expression for the particular gene of interest between the treated sample and the untreated sample. Experiments were run in triplicate, and for all analyses a p-value of <0.05 was considered to be statistically significant.

#### microRNA

cDNA was generated by TaqMan MicroRNA Reverse Transcription kit (4366596, Applied Bioscience) following manufacturer's protocol. Primer/probe mixes specific for each microRNA (RNU6B, 4373381; hsa-miR-19a, 4373099; hsa-miR-27a, 4373287; hsa-miR-33, 4373048; hsa-miR-101, 4373159; hsa-miR-133b, 4373172; hsa-miR-337, 4373044; hsa-miR-424, 4373201) were obtained from Applied Biosystems (Foster City, CA). TaqMan Universal Master PCR Mix (4304437, Applied Biosystems) was used for all reactions. All experiments were performed in triplicate. The average value of the triplicate readings for each unknown was normalized to the corresponding value for U6 RNA. The samples were analyzed using the delta-delta Ct method of analysis [43]. The final value obtained was a measure of the fold change in gene expression for the particular gene of interest between the treated sample and the untreated sample. For all analyses a P-value of <0.05 was considered to be statistically significant.

### Chromatin immunoprecipitation (ChIP) analysis

One 15 cm tissue culture dishes were plated with 95% confluent MDA-MB-231 cells for each condition. Cells were treated with 5 μg/ml actinomycin D for 30 minutes in serum-free DMEM. As specified, cells were incubated with 20 mM NaCl or LiCl for an additional 24 hours. 37% formaldehyde solution was added to each plate for a final concentration of 1.5% and incubated at 37°C for 15 minutes. Plates were washed one time with PBS containing 0.125 M glycine and Complete Mini Protease Inhibitor Cocktail (11836153001, Roche), and then a second time with PBS plus inhibitor. Cells were collected and spun for at 2000 rpm, 4°C for 5 minutes. The pellet was resuspended in 1 ml SDS lysis buffer (1% SDS, 10 mM EDTA, 50 mM Tris-HCl, pH 8.1) with protease inhibitors. Cells were then sonicated using a 15 second on, 45 second off program 4 times consecutively; then diluted 1:10 in ChIP Dilution Buffer (0.01% SDS, 1.1% Triton X-100, 1.2 mM EDTA, 16.7 mM Tris-HCl, pH .1, 167 mM NaCl) plus protease inhibitor. The samples were then precleared overnight at 4°C with 75 μl Protein A/G Plus-agarose beads (sc-2003, Santa Cruz) supplemented with 3 μl 10 mg/ml Sonicated Salmon Sperm DNA (201190, Stratagene, La Jolla, CA) and 13 μl 1 mg/ml BSA. Samples were then incubated with 10 μg RNA Polymerase II (N-20) (sc-899, Santa Cruz) overnight, tumbling at 4°C. Add 40 μl Protein A/G Plus-agarose beads for 2 hours rotating at 4°C. Samples were spun at 1000 rpm for 1 minute, then washed once with each: Low Salt Immune Complex Wash Buffer (0.1% SDS, 1% Triton X-100, 2 mM EDTA, 20 mM Tris-HCl, pH 8.1, 150 mM NaCl), High Salt Immune Complex Wash Buffer (0.1% SDS, 1% Triton X-100, 2 mM EDTA, 20 mM Tris-HCl, pH 8.1, 500 mM NaCl), LiCl Immune Complex Wash Buffer (0.25 M LiCl, 1% Nonidet P-40, 1% deoxycholic acid, 1 mM EDTA, 10 mM Tris-HCl, pH 8.1), and TE buffer. Samples were eluted twice in 100 μl in elution buffer (1% SDS, 0.1 M NaHCO_3_) vortexing for 15 minutes at room temperature. 12 μl 5 M NaCl was added to eluate and incubated at 65°C overnight. Then 4 μl 0.5 M EDTA, 8 μl 1M Tris-HCl, pH 6.5 and 2 μl of 10 mg/ml proteinase K was added and incubated at 45°C for 1 hour. The samples were cleaned up using the QIAGEN PCR Clean up kit (QIAGEN). PCR was performed using TaKaRa Premix Ex Taq kit (RR039, TaKaRa, Otsu, Shiga, Japan). The final reaction contains: 1× Premix Ex Taq, 2.5 μM of each primer, 8% DMSO, and 20% processed DNA (or 20% of a 1:10 dilution of input). For amplification of GAPDH, forward: (5′-TACTAGCGGTTTTACGGGCG-3′) reverse: (5′-TCGAACAGGAGGAGCAGAGAGCGA-3′). For amplification of CDH11,

forward: (5′-AAAGCAAAAGGGAGGGAGA-3′)

reverse: (5′-AGGTACAAACCCCCTCTGCT-3′) primers were used.

### Immunoblotting

Cells were treated for the indicated times. Cells were rinsed once with PBS and lysed with sample buffer (2% SDS, 10% glycerol, 10 mM Tris, pH 7.5) containing 1 mM sodium orthovanadate, 0.05 M sodium fluoride, and Complete mini protease inhibitors (11836153001, Roche Applied Science, Indianapolis, IN). Cell lysates were boiled for 10 minutes. Protein concentration was determined with a Bio-Rad DC Protein Assay (500-0116, Bio-Rad, Hercules, CA). After SDS-poly acrylamide gel electrophoresis, proteins were transferred to Protran BA 83 Nitrocellulose (10402495, Germany). Membranes were blocked with 5% milk in Tris-Buffered Saline containing 0.1% Tween-20, and incubated with primary antibody overnight at 4°C and subsequently with HRP-labeled secondary antibody. Proteins were visualized with ECL chemiluminescent reagents (RPN2106, Amersham Biosciences, Piscataway, NJ) or SuperSignal West Femto (34095, Pierce biotechnology Inc., Rockford, IL) using X-ray films (Denville Scientific Inc., Metuchen, NJ).

### Luciferase Assay Analysis

Cells were plated at 2×10^5^ in a 12-well plate in growth medium. Cells were transfected in triplicate with 1.8 ug of *luciferase* plasmid DNA and 0.2 ug of pCMV-*Renilla* (Promega). For cells treated with anti-Dicer siRNA, 100 nM siRNA was transfected together with *Luciferase* and *Renilla* using Amaxa nucleofection and collected and analyzed 48 hours later. Cells at varying densities were transfected using Amaxa nuclefection and 1.67×10^5^ cells (low) or 6.25×10^5^ cells (high) were plated in triplicate in a 12-well plate. Cells treated with meBIO or BIO (1 μM) were transfected in triplicate using Fugene6 (Roche). 24 hours after transfection, cells were treated with either BIO or meBIO. 48 hours after treatment cells were collected. In all cases, cells were lysed using passive lysis buffer provided with the Dual-*Luciferase* Reporter Assay System (Promega). 20 μl of each sample was loaded in duplicate in a 96-well plate and analyzed using the Dual-*Luciferase* Reporter Assay System reagents (E1960, Promega). The plate was read on a Berthold MicroLumat Plus LB 96V (Germany) using WinGlow software (Berthold).

## Supporting Information

Figure S1 (0.15 MB PDF)

Figure S2 (0.01 MB PDF)
