# Supplementary material for: Post-Transcriptional Regulation of Cadherin-11 Expression by GSK-3 and β-Catenin in Prostate and Breast Cancer Cells
Source: PLoS One. 2009 Mar 10;4(3):e4797. doi: 10.1371/journal.pone.0004797 (PMC2650783; doi:10.1371/journal.pone.0004797)
Supplement: Figure S1 — (0.15 MB PDF) [file pone.0004797.s001.pdf]

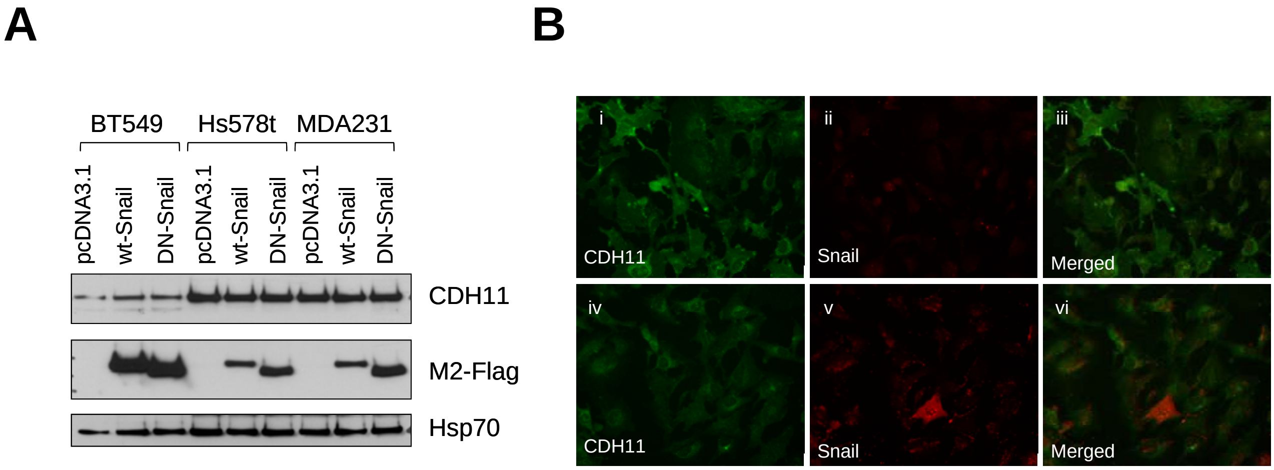

Figure S1: Snail fails to regulate cadherin-11 expression. A: Breast cancer cells, BT549, Hs578T, and MDA-MB-231 (MDA231), were plated at a medium density. Cells were transfected with pcDNA3, Flag-wt-Snail, or Flag-DN-Snail. 48 hours after transfection protein was collected for Western blot analysis. **B:** Hs578t breast cancer cells were transfected with either control vector (i-iii) or wild type Snail (iv-vi). 48 hours after transfection, the cells were immunostained for cadherin-11 (CDH11) or Snail.
