# Supplementary material for: Post-Transcriptional Regulation of Cadherin-11 Expression by GSK-3 and β-Catenin in Prostate and Breast Cancer Cells
Source: PLoS One. 2009 Mar 10;4(3):e4797. doi: 10.1371/journal.pone.0004797 (PMC2650783; doi:10.1371/journal.pone.0004797)
Supplement: Figure S2 — (0.01 MB PDF) [file pone.0004797.s002.pdf]

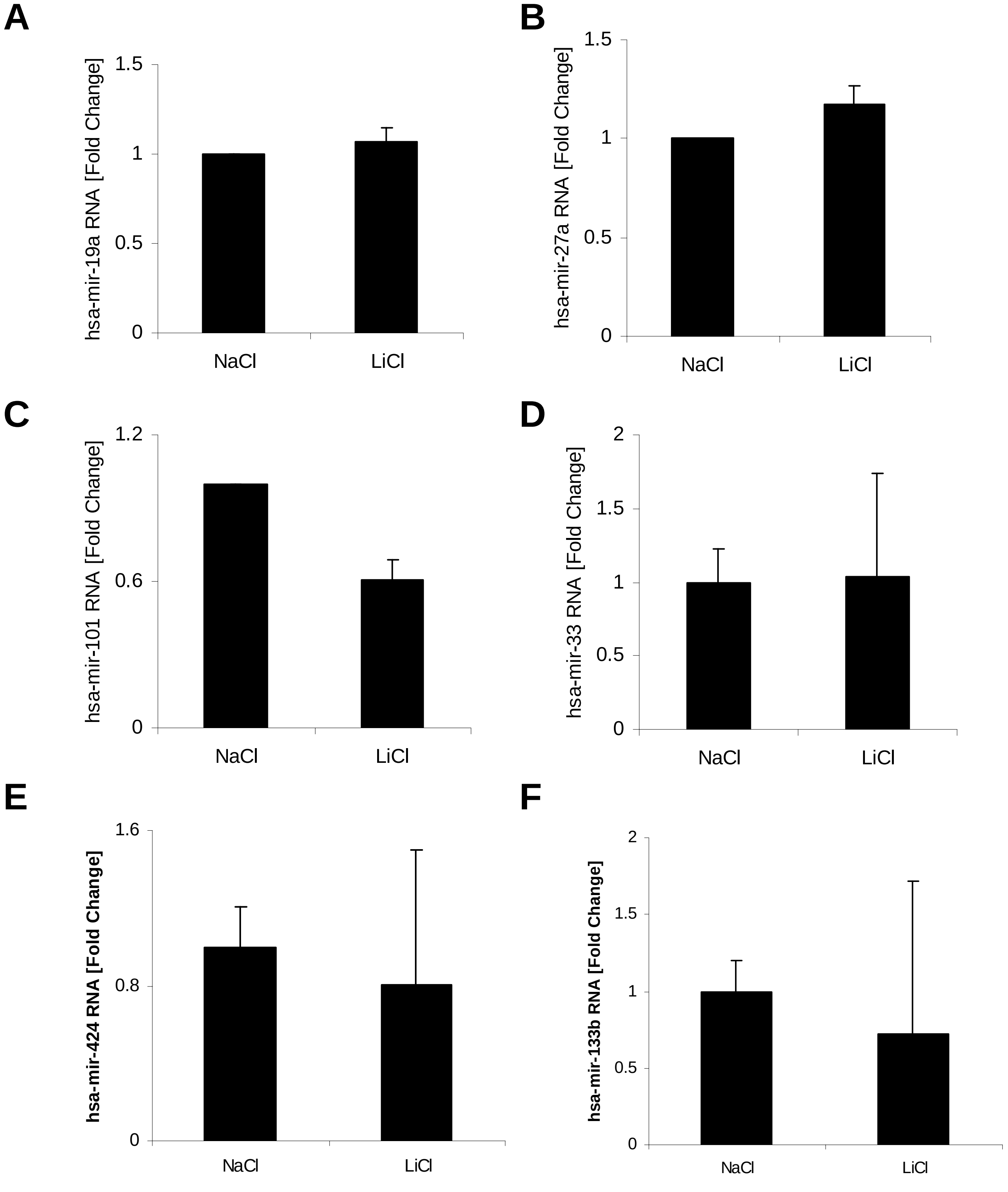

Figure S2: MicroRNAs predicted to bind to the cadherin-11 3'-UTR. A through F: MDA-MB-231 cells were treated with 20 mM LiCl or NaCl (control). 24 hours after treatment, total RNA was harvested for real-time PCR analysis.
